# Living on the edge: how philopatry maintains adaptive potential

**DOI:** 10.1098/rspb.2013.0305

**Published:** 2013-07-22

**Authors:** Victor A. Stiebens, Sonia E. Merino, Christian Roder, Frédéric J. J. Chain, Patricia L. M. Lee, Christophe Eizaguirre

**Affiliations:** 1 Department of Evolutionary Ecology of Marine Fishes, GEOMAR | Helmholtz Centre for Ocean Research, Kiel 24105, Germany; 2 National Institute for the Development of Fisheries (INDP), Mindelo 116, Cape Verde; 3 Turtle Foundation, Salrei, Boavista 411, Cape Verde; 4 Department of Evolutionary Ecology, Max Planck Institute for Evolutionary Biology, Ploen 24306, Germany; 5 Department of Biosciences, College of Science, Swansea University, Swansea SA2 8PP, UK; 6 School of Life and Environmental Sciences, Deakin University, Warrnambool, Victoria 3280, Australia

**Keywords:** philopatry, local adaptation, mitochondrial DNA, microsatellites, major histocompatibility complex, loggerhead sea turtle (*Caretta caretta*)

## Abstract

Without genetic variation, species cannot cope with changing environments, and evolution does not proceed. In endangered species, adaptive potential may be eroded by decreased population sizes and processes that further reduce gene flow such as philopatry and local adaptations. Here, we focused on the philopatric and endangered loggerhead sea turtle (*Caretta caretta*) nesting in Cape Verde as a model system to investigate the link between adaptive potential and philopatry. We produced a dataset of three complementary genomic regions to investigate female philopatric behaviour (mitochondrial DNA), male-mediated gene flow (microsatellites) and adaptive potential (major histocompatibility complex, MHC). Results revealed genetically distinct nesting colonies, indicating remarkably small-scale philopatric behaviour of females. Furthermore, these colonies also harboured local pools of MHC alleles, especially at the margins of the population's distribution, which are therefore important reserves of additional diversity for the population. Meanwhile, directional male-mediated gene flow from the margins of distribution sustains the adaptive potential for the entire rookery. We therefore present the first evidence for a positive association between philopatry and locally adapted genomic regions. Contrary to expectation, we propose that philopatry conserves a high adaptive potential at the margins of a distribution, while asymmetric gene flow maintains genetic connectivity with the rest of the population.

## 1. Introduction

Genetic diversity fuels species evolution as it is necessary for coping with changing environments [1] but is often impaired in endangered species [2]. Examples of endangered species with low genetic diversity are widespread, ranging from coelacanths [3] to marsupials [4]. In small populations, the adaptive potential rapidly declines with drift and inbreeding [5]. The adaptive potential is the capacity of populations to adapt to environmental changes and is often measured in terms of genetic diversity [5–7]. Furthermore, adaptive potential may also be eroded by processes that create structure, which then decreases gene flow among populations. Philopatry is such a process.

Philopatry is the return of an individual to its natal place to reproduce and is a common life-history strategy found in both aquatic and terrestrial animals [8]. The evolutionary origin of philopatry is debated and may stem from the assurance of finding returning mates for reproduction [9], the assurance of suitable sites to raise young [10] and/or natural selection maintaining locally co-adapted gene complexes for survival and reproduction [11]. A consequence of philopatry is that it enhances the formation of population structure by reducing gene flow among groups of individuals breeding at geographically separated locations. The creation of these smaller independent breeding colonies thereby depletes the adaptive potential of a population as a whole, owing to the genetic diversity being more distributed among populations rather than within populations. This was confirmed experimentally with fragmented populations of *Drosophila melanogaster* particularly when exposed to increased temperatures [12]. Thus, philopatry coupled with a significant decrease in population size may accelerate the loss of co-adapted gene complexes [5]. The hypothesis that philopatry undermines the adaptive potential of endangered species seems compelling, but it raises some crucial questions. Precisely, what are the roles of neutral and adaptive evolution in the maintenance of genetic diversity in endangered species? How does philopatry actually affect adaptive genetic diversity and thus adaptive potential? And, finally, what is the evolutionary significance of philopatry if it reduces genetic diversity in small populations?

To tackle these questions, we used the endangered and philopatric loggerhead sea turtle (*Caretta caretta*) nesting in Cape Verde as a model system. Sea turtles are important models for understanding a wide variety of biological phenomena such as animal migrations [13,14], mating strategies [15] and conservation genetics [16] in addition to being the classic model for studying philopatric behaviour [17,18]. In some rookeries, female loggerhead turtles are capable of extraordinary natal homing behaviour. However, there is variation in the geographical specificity of this behaviour among populations and sea turtle species (from some tens of kilometres up to thousands of kilometres within one population) [19]. Gene flow across rookeries is thought to be maintained by males, which appear to have less fidelity to natal breeding locations and/or may mate opportunistically on route to natal breeding locations [20,21].

We used two different neutral markers to infer the role of demography and gene flow in the maintenance of genetic diversity: the maternally inherited mitochondrial DNA (mtDNA) control region allowed us to characterize female philopatric behaviour [22,23], whereas bi-parentally inherited microsatellites enabled us to track male philopatric behaviour and male-mediated gene flow [15,20]. Aside from neutral markers, we also needed a genetic indicator of adaptive potential. Adaptive genes are those that underlie traits responding to selective pressures [24]. Examples are rare, but breakthrough studies have revealed a direct link between parasite resistance and the genes of the major histocompatibility complex (MHC; [25–27]). Parasites and pathogens are ubiquitous and readily shape the phenotype distribution of their hosts by natural selection [28]. MHC genes are part of the vertebrates’ adaptive immune system and particularly, MHC class I molecules bind peptides derived from the proteasome of endocellular parasites (viruses, some bacteria and cancer cells) and present them on the cell surface where an immune response is initiated [29]. Importantly, it has been shown that selection by a given parasite results in the increase in frequency of only those alleles present in the population that confer resistance to this parasite [25]; hence, the standing genetic variation at MHC loci may be associated with local adaptation [30]. MHC genes are therefore a natural choice for markers in investigating the link between philopatry and adaptive genetic diversity.

Our study system in the archipelago of Cape Verde is the second largest nesting aggregation of loggerhead turtles in the Atlantic Ocean [31,32]. The vast majority of nesting activity occurs on the eastern island of Boavista (85–90% of total nesting), followed by much lower numbers in Sal and S. Nicolau and only sporadic nesting at the margin of the population's distribution such as at S. Vicente [33]. After nesting, female turtles migrate from Cape Verde to feeding grounds along the west African coast. Interestingly, this population exhibits a dichotomy in foraging strategy that is linked to body size, with neritic feeding by larger turtles and oceanic feeding by smaller turtles [34,35]. In terms of conservation, loggerhead sea turtles in the Cape Verde archipelago are not only threatened by poaching, fisheries bycatch and coastal development [32,36], but also disease outbreaks [37]. Recently, the Cape Verde rookery was shown to be genetically different from other Atlantic and Mediterranean rookeries [31] and thus vulnerable to the loss of unique diversity. This population is therefore ideal for our study as it is clearly at a risk of losing adaptive potential.

## 2. Material and methods

### (a) Sample collection

**Figure 1.**
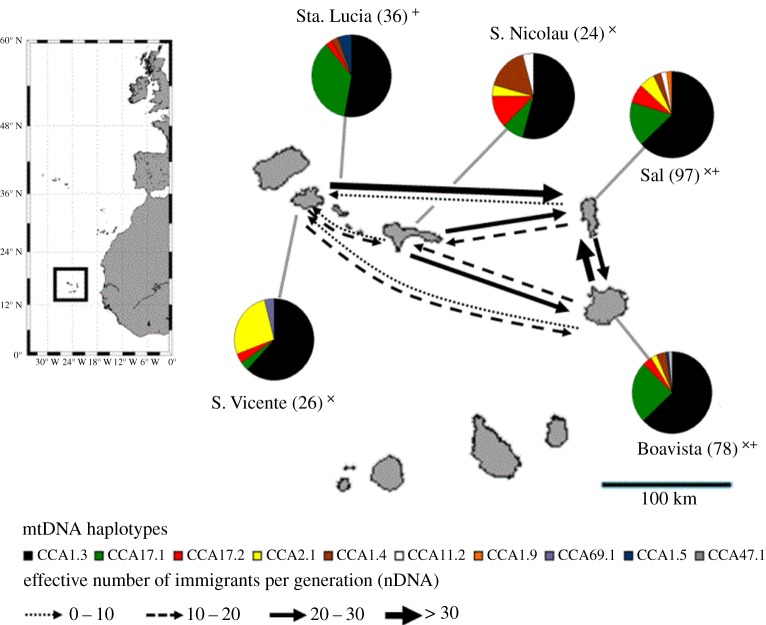
Pie charts representing mtDNA haplotypes (sample sizes) for the combined mtDNA dataset (x, this study; plus symbol, Monzón-Argüello *et al*. [31]). Arrows depict effective number of immigrants per generation calculated from microsatellite data across the nesting colonies. Note that sample sizes on nDNA do not correspond to numbers in brackets, because only the 2010 dataset was used here.

Tissue samples from 142 female loggerhead turtles were collected during the 2010 nesting season on four different islands of the Cape Verde Archipelago (Boavista, Sal, S. Nicolau and S. Vicente, see map in figure 1; GPS locations are in electronic supplementary material, table S1). Sampling of nesting females took place by carefully removing a 3 mm tissue sample from the non-keratinized skin of the flippers, using a single-use disposable scalpel (B. Braun, Tuttlingen, Germany). Turtles (*n* = 19) found dead (killed by poachers) were also sampled. In order to avoid duplicates in sample collection, nesting turtles were tagged with external metal Inconel tags (National Band and Tag Co., USA) on the front flippers and all carapaces of dead turtles were marked with paint. Samples were individually preserved in 96 per cent ethanol for later DNA analysis.

### (b) Molecular analyses

#### (i) DNA extraction

All tissue pieces were washed in distilled water for 1 min and then air-dried for 15 min. DNA extraction was performed using the DNeasy 96 blood and tissue kit (Qiagen, Hilden, Germany). DNA extraction failures mainly occurred in samples taken from turtles found dead on the beach (*n* = 26).

#### (ii) Mitochondrial control region, microsatellites and major histocompatibility complex amplification

All samples were amplified for an approximately 720 base pair (bp) fragment in the mtDNA control region (see the electronic supplementary material, table S1 for PCR composition, thermo-cycling protocol followed published methods using primers LCM15382 and H950 used in [31]). Resultant PCR products were then purified with ExoSAP-IT according to the manufacturer's protocol. Cycle sequencing from the forward direction (LCM15382) was performed using Big Dye Terminator v. 3.1 (Applied Biosystems, Darmstadt, Germany) and analysed with an ABI 3730 Genetic Analyzer (Applied Biosystems).

Eight polymorphic microsatellite loci were genotyped on an ABI 3130 Genetic Analyzer: Cc-10, Cc-17, Cc-22, Cc-16, Cc2 [38], 7C04, 2H12 and 2G10 ([39]; electronic supplementary material, table S2 for protocols).

The MHC class I exon 2 was sequenced on a 454 platform for the 142 sampled individuals, following Stiebens *et al*. [40]. Briefly, DNA concentrations were standardized to 10 ng µl^−1^. Then, for each individual, two independent PCRs were carried out using MHC-class-I-specific primers extended with 6 bp MID individual-based barcodes [40,41]. For each of the replicates, the amplification protocol was split into two steps with a reconditioning step to reduce PCR artefacts [42]. Afterwards, PCR amplicons were cleaned using Qiagen PCR purification kit (Qiagen). PCR concentrations were standardized and all amplicons were pooled and separated by electrophoresis on an agarose gel. The bands of expected sizes were cut, and the amplicons were extracted from the agarose using NucleoSpin extract II kit (Macherey-Nagel, Düren, Germany) before sequencing took place on a 454 platform.

### (c) Data and statistical analysis

#### (i) Mitochondrial control region

MtDNA control region sequences were aligned in CodonCode Aligner v. 3.5 (CodonCode Corporation) and then classified following the nomenclature of the Archie Carr Centre for Sea Turtle Research (ACCSTR). All new sequences were submitted to both ACCSTR and GenBank (accession numbers: KF021625 (CCA1.9) and KF021626 (CCA69.1)). A haplotype data file was created with the software dnasp v. 5.10.01 [43], and haplotype and nucleotide diversity were estimated [44]. To elucidate the evolutionary relationships among the different haplotypes, a network was generated in the software Network v. 4.6.1.0 [45].

To better understand female philopatric behaviour in Cape Verde and further increase statistical power, we combined our mtDNA dataset with one previously generated ([31], *n* = 128 individuals). For the islands where the datasets overlapped (Sal and Boavista), we computed *φ*_ST_ pairwise tests (50 000 permutations) and exact tests of population differentiation (Markov chain length was 500 000 with 10 000 dememorization steps) based on an expanded test analogous to the Fisher exact test [46,47] in Arlequin v. 3.1.5.2 [48]. Exact tests were performed because *φ*_ST_ values rely on Wright's island, model and the list of assumptions in this model are rarely met (i.e. equal subpopulation size, symmetric gene flow [49]). Because no differences were observed (Boavista: *φ*_ST_ = 0.021, *p* = 0.178, exact *p* = 0.621; Sal: *φ*_ST_ = 0, *p* = 0.607, exact *p* = 0.222), the two datasets were pooled. For the combined dataset, we then used *φ*_ST_ and the exact test to assess population structure across the entire sampled nesting range. Multiple testing was accounted for by applying the modified false discovery rate (FDR) threshold [50].

With the purpose of relating *φ*_ST_ values to geographical distances, a Mantel test was conducted using the vegan package of R v. 2.15.0 (R core Development Team). Geographical distances were estimated as the shortest possible swimming distance between islands using Google Earth (v. 5.2.1.1588). The relationship was tested using 10 000 permutations (Pearson's correlation), *φ*_ST_/(1−*φ*_ST_) and the log geographical distance between islands as suggested for an isolation by distance event in two dimensions, when using *F*_ST_s [51].

#### (ii) Microsatellites

Microsatellite alleles were called in GeneMarker v. 1.91 (Softgenetics LLC, State College, PA), and the data were imported into Arlequin v. 3.1.5.2 to estimate departure from Hardy–Weinberg equilibrium, observed and expected heterozygosity (*H*_o_, *H*_e_) and the mean number of alleles over all loci.

Pairwise *F*_ST_ and exact tests (same parameters as stated above) were also computed in Arlequin, and multiple testing was accounted for with the modified FDR method. Isolation by distance was assessed with Mantel tests in the same manner as previously mentioned, replacing *φ*_ST_ with *F*_ST_. Estimates of *F*_ST_ assume symmetric migration rates and equal population sizes, however, these assumptions are rarely met in natural populations [49]. Therefore, a maximum-likelihood method based on a coalescent approach [52] was implemented to calculate the effective number of immigrants per generation (ENI) across nesting colonies/nesting population ( = turtles nesting at the different islands, see §4) using Migrate v. 3.2.17. ENI was obtained by multiplying asymmetrical mutation-scaled immigration rates (*M*_donor population, receiving pop._) with the mutation-scaled effective population sizes (*Θ*_receiving pop._) for each possible migration across nesting islands [53]. Computations for *M* and *Θ* were performed using the settings modified after Bowen *et al*. [23]. Five replicates were performed, and means were calculated. ENI was then correlated to the geographical distance, taking into account the direction of the migration between the islands in a gradient from east to west and vice versa (gradient: Boavista–Sal–S. Nicolau–S. Vicente). To this end, an ANCOVA on log (ENI), including log (geographical distance), direction of the gene flow and their interaction as independent variables was conducted in the software R (http://www.r-project.org).

#### (iii) Major histocompatibility complex

Individuals harboured up to four different alleles suggesting the presence of up to four different loci [40], preventing us from using traditional analytical methods such as *F*_ST_. Even though alleles may originate from different duplicated loci, for the sake of simplicity, we named the different sequence variants ‘alleles’ (All MHC sequences are deposited on GenBank with accession numbers: KF021627 to KF021666).

The mean number of alleles per individual were not normally distributed (Shapiro–Wilk test: *W* = 0.868, *p*-value = 0), thus variation among nesting colonies was tested with a Kruskal–Wallis rank sum test.

Genetic divergence at MHC loci among nesting colonies was assessed using the R statistic computed through an analysis of similarity based on Bray–Curtis similarity matrix in primer v. 6 [54]. FDR correction for multiple testing was also applied. Additionally, we ran a permutation-based percentage test analysis (SIMPER) in order to estimate the contribution of each individual allele to the overall adaptive divergence [25].

To further understand the genetic structure at MHC, we first tested for the neutral role of isolation by distance in MHC divergence using a Mantel test between R statistics and log (geographical distance). To further control for a possible effect of geographical distance, we conducted a partial Mantel test with the R statistic correlated against the log (geographical distance), whereas controlling for the neutral divergence represented through *F*_ST_. All statistical tests were computed in the software R.

## 3. Results

### (a) Mitochondrial DNA: signs of female philopatry

In 133 turtles, eight distinct mtDNA control region haplotypes (717 or 723 bp) were found (figure 1 and electronic supplementary material, tables S3 and S4, and figure S5). Haplotypes showed overall low nucleotide divergence except for the CCA2.1 haplotype, which differed in 32 point mutations from the closest haplotype (see the electronic supplementary material, figure S5). This haplotype was more frequent in turtles nesting at the margin of the population distribution in S. Vicente (figure 1). Pairwise genetic tests showed high and significant levels of genetic differentiation among nesting islands (global exact test, *p* = 0.001). In particular, exact tests revealed that the population structure was more pronounced in turtles nesting in the west: the further west the turtles nested, the more pairwise comparisons were significant (S. Vicente (four significant tests), Sta. Lucia (three), S. Nicolau (two), Sal and Boavista (one); table 1*a*). Although slightly weaker, this gradient remains even after correction for multiple testing (table 1*a*). Pairwise *φ*_ST_ tests revealed similar structure arising from turtles nesting at the most northwestern island of S. Vicente (table 1*a*). It is noteworthy that the observed structure did not arise from isolation by distance (Mantel statistic *r* = 0.207, *p* = 0.340).

**Table 1.** Differentiation tests across nesting colonies: (*a*) *mtDNA:* pairwise *φ*_ST_ values (above diagonal) and *p*-values of exact tests of population differentiation (below diagonal). (*b*) *Microsatellites:* pairwise *F*_ST_ values (above diagonal) and *p*-values of exact tests of population differentiation (below diagonal). (*c*) *MHC class I:* pairwise R statistic values (above diagonal) and the corresponding *p*-values (below diagonal).

	Boavista	Sal	S. Nicolau	Sta. Lucia	S. Vicente
(*a*) *mtDNA:* exact *p\*φ**_ST_					
Boavista (**n** = 78)	—	0.002	0	0	**0.261**^a^
Sal (**n** = 97)	0.367	—	0	0.027	**0.162**^a^
S. Nicolau (**n** = 24)	**0.034**	0.172	—	0.016	**0.134**
Sta. Lucia (**n** = 36)	0.604	**0.046**	**0.008**^a^	—	**0.268**^a^
S. Vicente (**n** = 26)	**0.001**^a^	**0.035**	**0.021**	**0**^a^	—
(*b*) *msats:* exact *p*\*F*_ST_					
Boavista (**n** = 21)	—	0	0.004		**0.025**^a^
Sal (**n** = 40)	0.167	—	0		**0.009**
S. Nicolau (**n** = 24)	0.117	1.000	—		**0.009**
S. Vicente (**n** = 26)	**0.033**	0.265	0.232		—
(*c*) *MHC: p*\*R*-statistic					
Boavista (**n** = 26)	—	0.005	0.034		**0.063**^a^
Sal (**n** = 40)	0.354	—	0.029		0.014
S. Nicolau (**n** = 23)	0.085	0.103	—		0.002
S. Vicente (**n** = 23)	**0.017**^a^	0.261	0.414		—

^a^Bold values indicate statistical significance (*α* < 0.05). Depicts statistical significance after correction for multiple testing using the false discovery rate (FDR). Values in brackets represent sample sizes. Note that for (*a*), the dataset was combined with a previous study [31].

### (b) Microsatellites: males mediate gene flow

We genotyped eight microsatellite loci for 111 nesting turtles from four different islands (referred to as four nesting colonies, see §4 and electronic supplementary material, table S3). Diversity indices showed identical levels of variability across nesting colonies (see the electronic supplementary material, table S6). However, levels of observed heterozygosity increased in an eastward gradient (see the electronic supplementary material, figure S7).

The global exact test also revealed significant genetic structure across nesting colonies (*p* < 0.001). Pairwise exact tests suggested significant differences between the most geographically distant islands (Boavista and S. Vicente); however, none of the exact tests was significant after FDR correction for multiple testing (table 1*b*). On the contrary, pairwise *F*_ST_ revealed clear structure with low but significant *F*_ST_ values (ranging from 0.009 to 0.025) between the most distant sampled nesting colonies, even after correction for multiple testing (table 1*b*). Surprisingly, for such a large migratory species, this pattern suggests significant reproductive isolation by distance (Mantel test, *r* = 0.487, *p* = 0.040).

**Figure 2.**
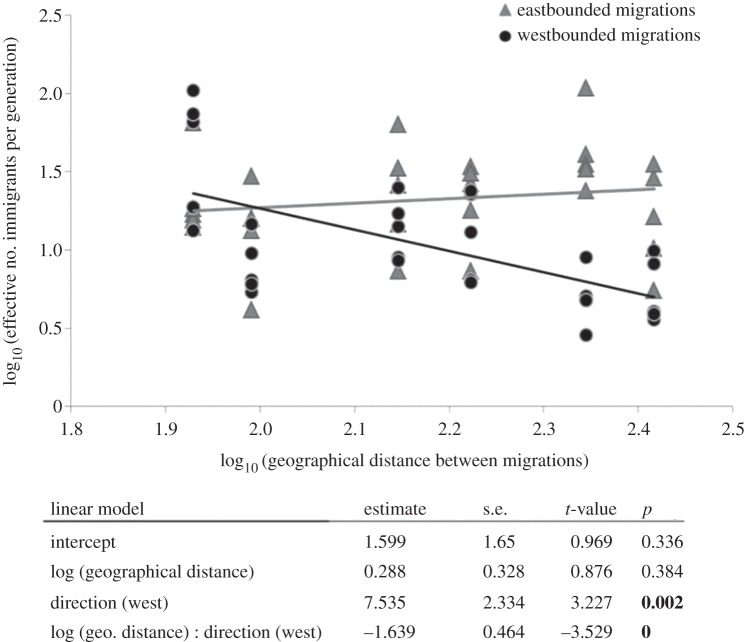
Relationship between effective number of immigrants per generation (log_10_-transformed) and geographical distance (log_10_-transformed) between migration routes, split for migrations in east- and westward directions. Statistical output of linear model is given in the table below the plot, with numbers in bold representing significance (*α* = 0.05).

In investigating whether gene flow was directional, we found that the effective number of immigrants per generation (ENI) was correlated to the direction of the migration, with a higher rate of migrations towards the east (ANCOVA, *t* = 3.227, *p* = 0.002, figure 2; electronic supplementary material, table S8). Furthermore, ENI was significantly correlated with an interaction between the direction of the gene flow and the geographical distance: in a westwards direction, ENI decreased with geographical distance, whereas in eastbound migrations, ENI remained stable at intermediate levels (ANCOVA, *t* = −3.529, *p* < 0.001, figure 2).

### (c) Major histocompatibility complex class I: signs of local adaptation

**Figure 3.**
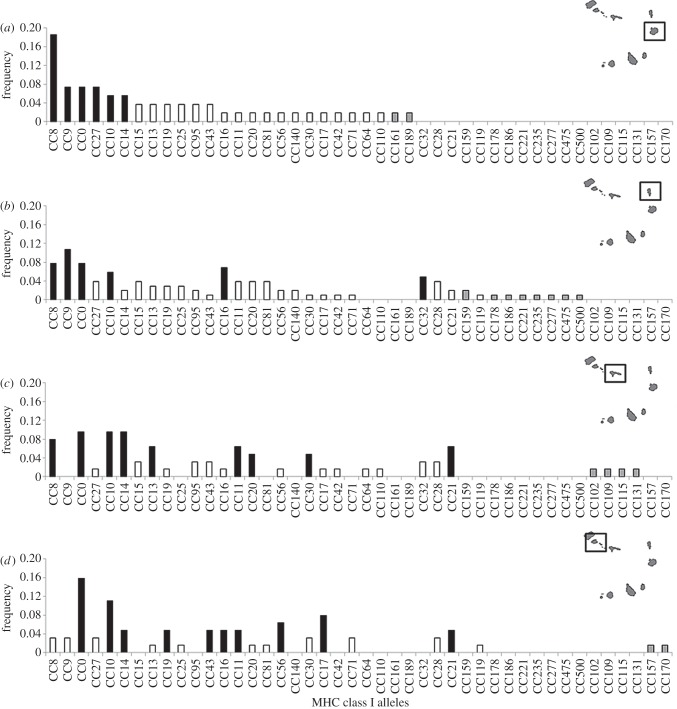
MHC class I allele abundances (proxies for frequencies in duplicated gene system) across nesting colonies. Alleles are ordered by decreasing frequency starting with turtles nesting in (*a*) Boavista followed by the islands of (*b*) Sal, (*c*) S. Nicolau and (*d*) S. Vicente. Black bars represent alleles with a frequency higher than 4%. Grey bars indicate alleles that are unique to a given island.

We sequenced a 216 bp long fragment of the MHC class I region in 112 individuals using 454 next-generation sequencing technology (see the electronic supplementary material, table S3). We detected 44 different variants (40 different amino acid sequences (see the electronic supplementary material, figure S9)) of which 16 (36.36%) were found to be unique to one specific island (figure 3). Genetic variability at this MHC locus in terms of the mean number of alleles per individual was similar across nesting islands (Kruskal–Wallis *χ*²_3,112_ = 6.959, *p* = 0.073).

MHC class I allele frequency distributions were different between the most distantly separated islands, which remained significant even after correction for multiple testing (Boavista and S. Vicente, table 1*c* and figure 3). The permutation-based percentage test analysis (SIMPER) demonstrated that two alleles mainly accounted for a cumulative divergence of 21.44 per cent (allele CC0 11.82% and allele CC8 9.62%).

Then, we tested whether the observed differences among allele pools were simply due to the distance between islands: the Mantel test correlating MHC divergence with geographical distance revealed no statistically significant association (*r* = 0.835, *p* = 0.084), suggesting an adaptive pattern of MHC divergence. Additionally, a partial Mantel test of the MHC divergence against geographical distance while controlling for neutral genetic structure represented through *F*_ST_ indicated no significant correlation (*r* = 0.770, *p* = 0.15), further confirming that the observed genetic structure was not solely a result of neutral drift.

## 4. Discussion

Contrary to our original hypothesis, philopatry as well its associated reduction of gene flow, does not deplete genetic diversity, but rather maintains unique genetic diversity for the whole population. We suggest that this effect may be particularly dramatic at the margins of species’ distribution, because populations there can support genetic innovation at a higher rate [55,56].

Even though distances between islands of this oceanic rookery only ranged from 80 to 260 km, we found a clear pattern of genetic structure of mtDNA haplotypes among nesting islands (figure 1 and table 1*a*), consistent with a high accuracy of female philopatry as reported for other rookeries [19,57]. This island-specific behaviour was strong in the western part of the distribution range, which was supported by both high *φ*_ST_ and exact tests for S. Vicente turtles. The slightly weaker genetic structure in the east was consistent with the much higher nesting densities there [33]. Non-philopatric ‘explorative behaviours’ are needed to colonize new nesting environments on evolutionary time scales [58]. Thus, a high number of turtles in the east causes a proportionally higher number of explorative behaviours, resulting in a less distinct genetic structure in the east [59]. Nonetheless, our data suggest that Cape Verde supports multiple genetically distinct nesting colonies, contrary to what was previously thought [31]. The difference probably stems from an extended sampling scheme in this study, which also included nesting locations that had not been previously sampled (figure 1).

Another striking discovery was that genetic structure based on the bi-parentally inherited microsatellites, unlike that of the maternally inherited mtDNA, followed a pattern of isolation by distance. This pattern of isolation by distance revealed the possible existence of male philopatry to specific mating grounds. Our data support the existence of male philopatry at least at a regional scale (e.g. east versus west). Nevertheless, the observed pairwise comparisons at nuclear markers appeared lower than for that of the mtDNA control region. Although this pattern may arise from slower allelic fixation of microsatellites (although mtDNA in sea turtles evolves at a slower pace than in other vertebrates [60]) and a fourfold higher effective population size of nDNA compared with mtDNA [61], lower levels of nuclear differentiation in sea turtles are generally thought to arise from male-mediated gene flow through opportunistic mating [23]. This was also supported in our dataset by the more sophisticated test of asymmetric gene flow, where we detected a clear pattern of isolation by distance in a westwards gradient compared with a constant gene flow in an eastward direction (figures 1 and 2). Such a pattern suggests a scenario in which before mating, male loggerheads are likely to first arrive at the eastern edge of the archipelago from the direction of west African feeding grounds. Only the males with fidelity to natal areas at the most western edge of the archipelago would mate at those relatively far locations, but the consequence of opportunistic mating as all males return eastwards to their feeding grounds would be a high asymmetrical gene flow towards the east. While this speculative scenario may explain our current findings, it requires further testing, such as with tracking experiments.

Our original hypothesis stated that philopatry in endangered species would deplete the overall adaptive potential of the rookery because of reduced gene flow and smaller nesting colony sizes. Contrary to that expectation, the genetic diversity at MHC loci was not low: each nesting colony displayed more than 20 MHC class I alleles, which, compared with other endangered species seems to be high (our dataset: total of 44 MHC class I alleles (*n* = 112); Namibian cheetah: 10 MHC class I alleles (*n* = 108) [62]; Tasmanian devil: 25 MHC class I alleles (*n* = 387) [63]; European bison: seven MHC class alleles (*n* = 99) [64], but see also Bengal tiger: 14 MHC class I alleles (*n* = 14) [65]). It is interesting that the observed MHC diversity was locally structured at the most distant nesting colonies, even though loggerhead sea turtles spend almost their entire adulthood in common feeding grounds off the west coast of Africa [34]. To confirm the independence of the genetic structure from the feeding grounds, we show that no relationship exists between genetic structure and turtle sizes (see the electronic supplementary material, table S10) with size being a good indicator of foraging strategy (neritic versus oceanic) for Cape Verde loggerhead turtles [34,35]. This reinforces our conclusion that the genetic structure found at the nesting colonies did not arise from clustering by the feeding grounds, but from philopatry. The fact that the structure for adaptive markers was strongest where neutral genetic structure was also greatest allowed us to conclude that the structure arising from philopatry maintains the isolation of local pools of MHC alleles. Multiple non-exclusive reasons could explain this tight link. On the one hand, MHC diversity could be the result of neutral processes, whereas, on the other hand, MHC diversity could be shaped by natural selection. Evidence that natural selection may be acting is twofold: first, microsatellites revealed genetic differences between the western and the eastern turtles and strong gene flow from the west to the eastern colonies. Therefore, we found a higher genetic diversity in the east than in the west, as demonstrated by a higher observed heterozygosity than expected in the eastern island of Boavista (see the electronic supplementary material, figure S7). Under a ‘no selection’ scenario, MHC diversity should display the same pattern. However, this was not the case, as turtles nesting on Boavista displayed the lowest MHC diversity (mean number of alleles) and lowest divergent allele pools (figure 3). Second, partial Mantel tests accounting for geographical distances failed to correlate MHC divergence with neutral divergence, further suggesting the independence of neutral and selective processes on the MHC diversity in the Cape Verde rookery.

These results allow us to conclude that despite high gene flow, selection has contributed to sort different MHC alleles among nesting colonies. To the best of our knowledge, this represents the first evidence for an association between philopatry and locally adapted genomic regions. We therefore propose the alternative hypothesis that philopatry may be acting to maintain a high adaptive potential in sea turtles by facilitating the retention of locally adapted genetic polymorphism. Although we cannot clearly point out how selection occurs, it may be possible that there are differences in the incubation environment, which include factors such as parasites (here understood in its wider sense of bacteria, virus, fungus, etc.).

Our results also emphasize the synergistic interaction of asymmetric gene flow and the maintenance of genetic diversity in a philopatric species. One remarkable discovery was that the smallest nesting colony at the extreme westward margin held the most differentiated set of MHC alleles (figure 3 and table 1c). Increased genetic differentiation at the periphery of populations is indeed a common observation for both plants and animals and a central concept in theories about the evolution of species ranges [66], but gene flow from the more abundant centre to the edge of a range is expected to counteract the benefits of local adaptation [67]. Here, philopatric behaviour has allowed the evolution of differentiated MHC allele pools, and fortuitously, asymmetric gene flow away from the western edge has prevented genetic swamping of the most marginal colony. The asymmetric gene flow towards the more abundant colonies in the east has further consequences for the maintenance and spread of adaptive potential. For instance, it would allow for beneficial MHC alleles conserved at S. Vicente (west) to rapidly sweep/introgress through the population in the face of an attack from a particular disease/parasite or a drastic change in the environment [30].

In the scope of conservation biology, the Cape Verde rookery should not be considered as a single population but rather various nesting colonies that harbour important genetic variation necessary for future adaptations, especially in the face of climate change and the maintenance of a healthy metapopulation. We uphold the suggestion that marginal colonies should not be neglected as inconsequential components of a population [68] and in support, we have provided empirical evidence to demonstrate that the edges of a population may instead be important reserves of unique variation and contribute disproportionally to the adaptive potential and future viability of that population.

In summary, we showed that local immunogenetic adaptation may be a driver for the evolution of philopatry and that philopatry in the endangered loggerhead turtles maintains the adaptive potential of the species. Furthermore, we showed that the edges of populations should be considered as an important reservoir of genetic diversity, particularly in the face of current rapid global changes.
